# Diversification processes between monogenoids (Dactylogyridae) and their marine catfish (Siluriformes: Ariidae) from the Atlantic coast of South America

**DOI:** 10.1017/S0031182022001615

**Published:** 2023-02

**Authors:** Geusivam B. Soares, Edson A. Adriano, Marcus V. Domingues, Juan Antonio Balbuena

**Affiliations:** 1 Departamento de Biologia Animal, Instituto de Biologia, Universidade Estadual de Campinas (UNICAMP), Rua Monteiro Lobato, 255, CEP 13083–862 Campinas, São Paulo, Brazil; 2 Departamento de Ecologia e Biologia Evolutiva, Universidade Federal de São Paulo (UNIFESP), Rua Professor Arthur Riedel, 275, Jardim Eldorado, CEP 09972–270, Diadema, São Paulo, Brazil; 3 Instituto de Estudos Costeiros, Universidade Federal do Pará (UFPA), Travessa Leandro Ribeiro, s/n, Aldeia, CEP 68600–000, Bragança, Pará, Brazil; 4 Institut Cavanilles de Biodiversitat i Biologia Evolutiva, Universitat de València, València, Spain

**Keywords:** Brazilian coast, coevolution, cophylogenetic signal, cophylogeny, Monogenoidea, Neotropical region

## Abstract

Due to their high specificity, monogenoids from fish provide an interesting model to study historical associations of hosts and parasites. High agreement between host and parasite phylogeny is often interpreted as evidence of cospeciation. However, cophylogenetic signal may also arise from other, either adaptive or non-adaptive, processes. We applied the recently developed Cophylospace Framework to better understand the evolutionary relationship between monogenoids and marine catfish from the Atlantic coast of South America. The associations between 12 marine catfish and 10 monogenoid species were assessed. Molecular data of host and parasite species were used for phylogenetic reconstruction. We used anchor morphology based on Procrustes coordinates to evaluate whether closely related hosts are associated with morphologically similar parasites. To assess the association between parasite phylogeny and host morphology, we produced a distance matrix based on morphological characters of catfishes. Agreement between phylogenies and between phylogeny and morphology was measured using Procrustes *R*^2^ computed with PACo. The parasite phylogeny obtained in this study represents the first complete phylogenetic hypothesis of monogenoids parasitizing ariids from South America. The Cophylospace analysis suggested that phylogenetic and morphological distance of monogenoids contributes similarly to explain the pattern of host–parasite associations, whereas parasite phylogeny is more strongly associated with the morphological traits of the hosts than with host phylogeny. This evidence suggests that cospeciation is not a major force accounting for diversification in the monogenoids studied. Rather host morphological traits seem to be a more important driver, which conforms with evidence from other host‒monogenoid systems.

## Graphical abstract

**Figure:**
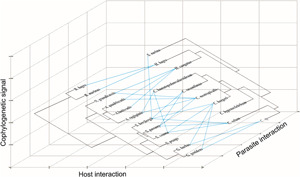

## Introduction

Reconstruction of the association between 2 or more lineages over evolutionary time has been a recurrent theme spanning different biological fields, from molecular evolution to coevolution and biogeography (Page, 2003). Host–parasite coevolution in particular has been the subject of numerous studies for decades. The common goal of such efforts has been estimating the joint evolutionary history that gave rise to the extant patterns of association between hosts and parasites (Klassen, 1992; Desdevises, 2007; Lei and Olival, 2014). Since the advent of molecular genetics and phylogenetic reconstruction, coevolutionary studies have advanced mainly through the application of cophylogenetic analyses (Poulin, 2021).

In fact, a variety of analytical tools has been developed for cophylogenetic studies in the last decades (Drinkwater and Charleston, 2016; Hutchinson *et al*., 2017; Balbuena *et al*., 2020). These methods have been useful to reveal a range of cophylogenetic patterns, but identifying the processes that give rise to them remains challenging (Althoff *et al*., 2014; Blasco-Costa *et al*., 2021). It is important to note that cospeciation patterns do not necessarily provide evidence for host–parasite coevolution, as they may indeed result from different processes, such as vicariance, phylogenetic tracking, vertical transmission or coevolution [see Althoff *et al*. (2014) for definitions of these processes]. Building on this idea, Blasco-Costa *et al*. (2021) proposed a new ‘Cophylospace Framework’ aimed at obtaining better insight into the mechanisms that drive coevolutionary processes.

This new Cophylospace Framework is defined in a 3-dimensional space based on 3 quantitative parameters: cophylogenetic signal, parasite interaction and host interaction. Cophylogenetic signal measures the agreement between the host and parasite histories. This is assessed by quantifying the degree of phylogenetic congruence between hosts and parasites. The interaction parameters are meant to measure the relationship between phylogeny of one of the symbiotic partners and morphological similarities between the other partner. These interactions are expected to reflect the degree of phylogenetic tracking of one partner over the other. Thus, a given cophylogenetic scenario could be explained in terms of its position along the 3 axes, reflecting the relative contributions of different processes (see Blasco-Costa *et al*., 2021, Fig. 1, p. 6). For example, significant congruence between host and parasite phylogenies could be taken as evidence for cospeciation being important in the system studied. However, cospeciation can result from 4 different mechanisms: coevolution, vicariance, phylogenetic tracking or vertical transmission. For instance, if the relationship between parasite phylogeny and host morphology was stronger than between the phylogenies of hosts and parasites, this suggests that parasite speciation is mostly determined by adaptation to diversification of host resources. Thus, phylogenetic tracking rather that strict host–parasite cospeciation is likely the mechanism accounting for the patterns observed. Nevertheless this framework remains to be tested in different host–parasite systems.

In aquatic environments, parasitic platyhelminths from the Class Monogenoidea and their fish hosts have attracted much attention in historical reconstruction studies. This is mainly due to their generally high host specificity which promotes studies aimed at establishing relationships between the ecological characteristics of the hosts and the diversity of their parasites (Rohde, 1979; Sasal *et al*., 1998; Poulin, 2002; Míguez-Lozano *et al*., 2017). However, although high host specificity can result from cospeciation (Noble *et al*., 1989; Kearn, 1994), it can also arise from other adaptive and non-adaptive processes (Boeger and Kritsky, 1997; Braga *et al*., 2014). In recent years, different studies of monogenoids and their fish hosts have suggested that host switching and duplication are the most recurrent evolutionary events in parasite diversification (Desdevises *et al*., 2002; Šimková *et al*., 2004, 2006, 2013; Domingues and Boeger, 2005; Huyse and Volckaert, 2005; Mendlová *et al*., 2012; Hahn *et al*., 2015; Vanhove *et al*., 2015; Míguez–Lozano *et al*., 2017; Graça *et al*., 2018; Rahmouni *et al*., 2022; Seidlová *et al*., 2022), whereas cospeciation is relatively rare (Desdevises *et al*., 2002; Mendlová *et al*., 2012; Míguez–Lozano *et al*., 2017; Graça *et al*., 2018; Rahmouni *et al*., 2022, Seidlová *et al*., 2022).

The monogenoid genera *Chauhanellus* Bychowsky & Nagibina, 1969, with 30 species, *Hamatopeduncularia* Yamaguti, 1953, with 32 and the monotypic *Susanlimocotyle* Soares *et al*., 2021 are related genera within the Dactylogyridae with all species recorded on the gills (or nostrils in the case of *Susanlimocotyle*) of marine catfish (Ariidae, Siluriformes) worldwide (Lim *et al*., 2001; Domingues *et al*., 2016; Illa *et al*., 2019; Soares *et al*., 2021; Soo and Tan, 2021). The distribution and colonization of these species may have been influenced by the evolutionary history of the Ariidae. In fact, vicariance associated with the fragmentation of Gondwana seems the most plausible hypothesis to explain the current distribution of these monogenoids across the different zoogeographic regions (i.e. Neotropical, Nearctic, Palearctic, Ethiopian and Oriental) (Lim *et al*., 2001; Betancur-R, 2009; Kritsky *et al*., 2009; Soares *et al*., 2021).

To date, only 6 species of *Chauhanellus*, 2 of *Hamatopeduncularia* and *Susanlimocotyle narina* Soares *et al*., 2021 have been recorded in South America (Domingues and Fehlauer, 2006; Domingues *et al*., 2016; Soares *et al*., 2021). However, our ongoing studies indicate that the actual number of species in the region is probably much higher.

Most studies to date have suggested that the recent diversification of ariids on the Atlantic coast of South America is mostly associated with adaptive processes linked to their occupation of different environments (Marceniuk, 2005; Marceniuk *et al.* 2012*a*, 2017, 2019a, 2022; Da Silva *et al*., 2016). Thus, this dactylogyrids–marine catfish association is extremely attractive for biogeographic and coevolutionary studies, since it is reasonable to expect that these monogenoids will accommodate evolutionary events associated with their hosts (Soares *et al*., 2021). Herein, we apply the Cophylospace Framework to this host–parasite system. Our aim is to reconstruct molecular phylogenies for the hosts (Ariidae) and their monogenoid parasites to assess which evolutionary process has likely accounted for the diversification of the dactylogyrid species on their ariid hosts. Based on evidence from previous studies, we hypothesize that monogenoid diversification is mostly likely driven by phylogenetic tracking of host resources rather than by strict host–parasite cospeciation.

## Materials and methods

### Study area, host and parasite collection

**Fig. 1.**
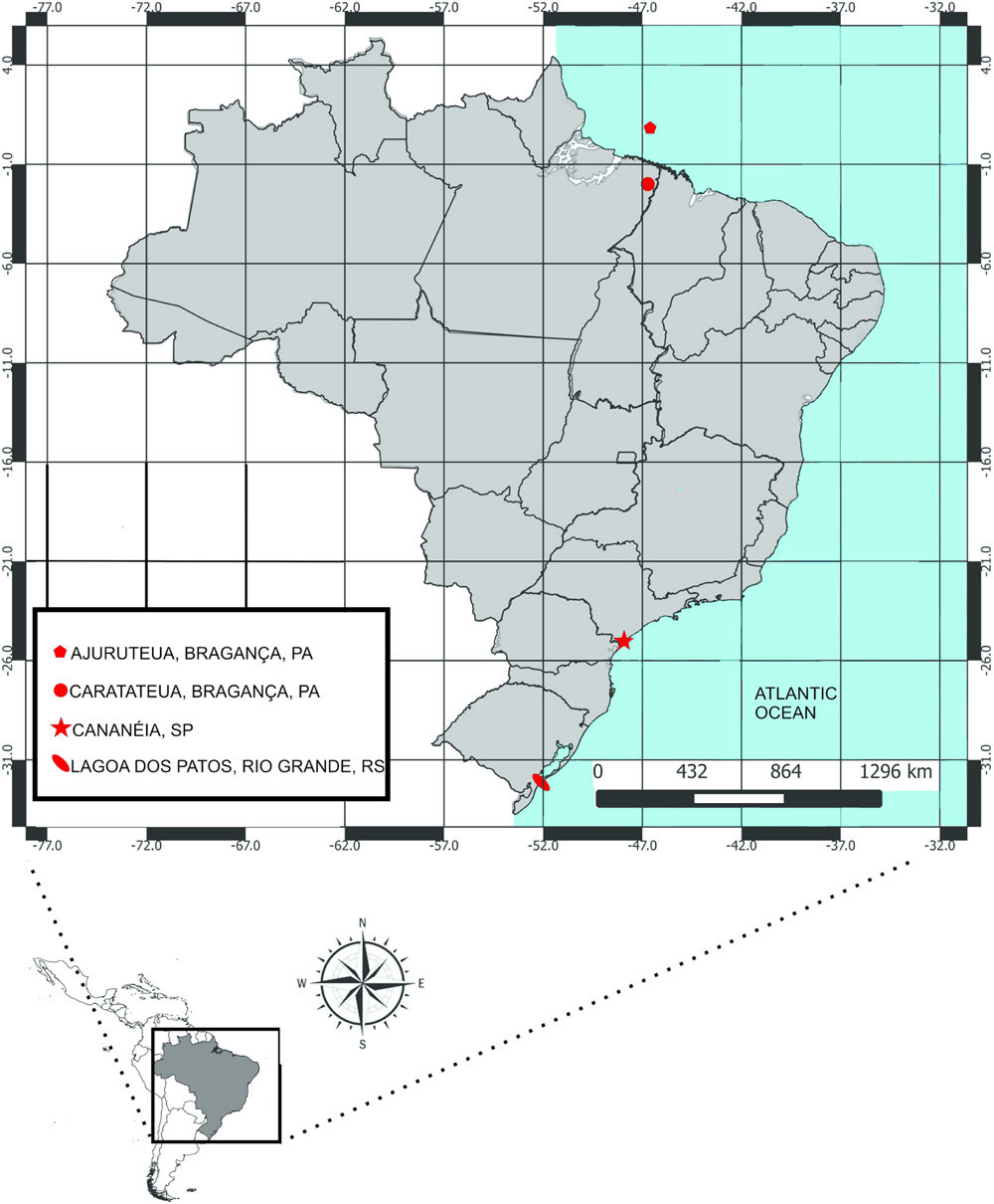
Sampling sites of fish in the Atlantic coast of Brazil.

Fish were collected by local fishermen with trammel net and hooks from 4 localities along the Brazilian coast (Table 1, Fig. 1). Eleven species belonging to the family Ariidae were collected: *Amphiarius rugispinis* (Valenciennes, 1840), *Aspistor luniscutis* (Valenciennes, 1840), *Aspistor quadriscutis* (Valenciennes, 1840), *Bagre bagre* (Linnaeus, 1766), *Genidens barbus* (Lacepède, 1803), *Genidens genidens* (Cuvier, 1829), *Notarius grandicassis* (Valenciennes, 1840), *Sciades couma* (Valenciennes, 1840), *Sciades herzbergii* (Bloch, 1794), *Sciades passany* (Valenciennes, 1840), *Sciades proops* (Valenciennes, 1840) (Table 1). The host species were chosen according to previous records of *Chauhanellus* spp., *Hamatopeduncularia* spp. and *S. narina* Soares *et al*., 2021, in the study area (Domingues and Fehlauer, 2006; Domingues *et al*., 2016; Soares *et al*., 2021). The data on the species *Bagre marinus* (Mitchell, 1815) used in the analyses were based on the literature (Mendoza-Franco *et al*., 2018). Host scientific names follow Marceniuk *et al*. (2012*b*).

**Table 1.** Monogenoid species included in the present study

Parasite	Host	Site^a^	Locality^b^
*Chauhanellus boegeri* OP681529^c^	*Genidens barbus*	G	Cananéia (25°02′09.2″S;47°54′57.8″W), SP, BR
*Chauhanellus neotropicalis* OP681530^c^	*Aspistor luniscutis*	G	Cananéia (25°02′09.2″S;47°54′57.8″W), SP, BR
*Chauhanellus hypenocleithrum* OP681533^c^	*Sciades proops*	G	Ajuruteua (0°49′31″N;46°36′29″W), Bragança, PA, BR
*Chauhanellus susamlimae* OP681527^c^	*Sciades passany*	G	Caratateua (1°59′41.91″S; 46°43′21.385″W), Bragança, PA, BR
*Chauhanellus hamatopeduncularoideum* OP681531^c^	*Amphiarius rugispinis*	G	Ajuruteua (0°49′31″N;46°36′29″W), Bragança, PA, BR
*Chauhanellus velum* OP681528^c^	*Sciades herzbergii*	G	Ajuruteua (0°49′31″N;46°36′29″W), Bragança, PA, BR
*Chauhanellus* sp. OP681534^c^	*Genidens barbus*	G	Lagoa dos Patos (32°08′05.7″S; 52°06′11.2″W), Rio grande RS, BR
*Susanlimocotyle narina* OP681525^c^	*Sciades herbergii*	N	Ajuruteua (0°49′31″N;46°36′29″W), Bragança, PA, BR
*Hamatopeduncularia bagre* OP681526^c^	*Bagre bagre*	G	Ajuruteua (0°49′31″N;46°36′29″W), Bragança, PA, BR
*Hamatopeduncularia cangatae* OP681532^c^	*Aspistor luniscutis*	G	Cananéia (25°02′09.2″S;47°54′57.8″W), SP, BR

Host species, infestation site, locality (geographical coordinates) and GenBank accession numbers are provided.

a G, gills; N, nasal cavities

b SP, São Paulo; PA, Pará; RS, Rio Grande do Sul; BR, Brazil

c GenBank accession numbers of the DNA sequences of genes *18S rDNA*, *ITS1*, *5.8S rDNA* and *ITS2*.

Gills and nasal cavities of fishes were examined for monogenoids following Soares *et al*. (2019) for morphological study, and Soares *et al*. (2021) for molecular characterization. Each gill arch and nasal cavity were examined individually.

Each monogenoid specimen subjected to molecular analysis was divided using fine needles under a dissecting microscope. The anterior half of the body (without the male copulatory organ) was placed in a 1.5 mL microtube with 96% ethanol for genomic DNA extraction. The posterior part containing the haptoral complex and the male copulatory organ was flattened under coverslip pressure and mounted in Hoyer's solution for species identification. These fragments served also as vouchers [hologenophores sensu Pleijel *et al*. (2008)]. Identification to species was carried out according to Domingues and Fehlauer (2006), Domingues *et al*. (2016) and Soares *et al*. (2021). Hologenophores are deposited at the collection of Platyhelminthes of the Adão José Cardoso Museum of Zoology of the State University of Campinas (ZUEC PLA), São Paulo state, and the Invertebrate Collection of the Museu Paraense Emílio Goeldi (MPEG), Belém, Pará state, Brazil, respectively under No (ZUEC PLA 184–187 ; MPEG 276–278). The specimen, herein referred to as *Chauhanellus* sp., possibly represents a new species to science that will be described in due course.

### DNA extraction, PCR and sequencing, alignment and phylogenetic analyses

For parasites, total genomic DNA was extracted using Qiagen Dneasy^®^ Blood and Tissue Kits (animal tissue protocol), according to the manufacturer's protocol, with a final volume of 30 *μ*L. The DNA concentration was verified using a NanoDrop 2000 spectrophotometer (Thermo Fisher Scientific, Massachusetts, USA) at 260 nm.

Partial 18S rDNA, ITS1, 5.8S rDNA and ITS2 regions were amplified using a 2-round polymerase chain reaction (PCR). In the first round, DNA was amplified with the primer pair 1200F (Littlewood and Olson, 2001) and D2 (Wu *et al*., 2006). In the second round, for the semi-nested PCRs, the primer combinations were 1200F and 28SR1 (Wu *et al*., 2006), which amplified a fragment of ~1131 bp.

PCRs were performed in a Matercycler^®^ nexus (Eppendorf, Hamburg, Germany) with a final volume of 25:12.5 *μ*L of DreamTaq Green PCR Master Mix (2×) (Thermo Scientific, Wilmington, USA), following the manufacturer's recommendations, 0.5 mm of each primer and 3 *μ*L of the extracted DNA. The PCR profile was set as follows: an initial denaturation at 95°C was performed for 3 min, followed by 34 cycles of 94°C for 30 s, 56°C for 30 s, 72°C for 90 s and a final elongation at 72°C for 4 min. The semi-nested PCRs were conducted with 1 *μ*L of the PCR product, diluted 1:1 in ultrapure water, applying the same cycling conditions. Amplicons were electrophoresed on 2% agarose gel in a TAE buffer (Tris 40 mm, acetic acid 20 mm, ethylenediaminetetraacetic acid 1 mm) stained with SYBRsafe^®^ (Invitrogen, Thermo Fisher Scientific) alongside a 1 kb Plus DNA Ladder (Invitrogen, Thermo Fisher Scientific) at 100 V for 30 min. PCR products were purified using a QIAquick PCR Purification Kit (Qiagen, USA) and sequencing was carried out with the BigDye^®^ Terminator v3.1 Cycle Sequencing Kit (Applied Biosystems™, California, USA) in a 3500 DNA sequencing analyser (Applied Biosystems) at Helixxa Company (Paulínia, São Paulo State, Brazil) or at the Human Genome Research Center (HGRC) of the University of São Paulo (São Paulo State, Brazil), using the primers pair 1200F and 28SR1 for amplification.

For the phylogenetic reconstruction of the hosts, *Cytb* and *RAG2* partial sequences were retrieved from GenBank (Table 2).

**Table 2.** GenBank accession numbers of the DNA sequences of genes *Cytb* and *RAG2* partial of fish hosts and associated species of *Chauhanellus*, *Hamatopeduncularia* and *Susanlimocotyle* detected in the present effort on each fish species

Hosts	*Cytb*	*RAG2*	Parasite species
*Amphiarius rugispinis*	AY688668	DQ990506	*Chauhanellus hamatopeduncularoideum*^a^ *Chauhanellus neotropicalis*
*Aspistor luniscutis*	FJ626172	JN672841	*Hamatopeduncularia cangatae*^a^ *Chauhanellus neotropicalis*^a^
*Aspistor quadriscutis*	AY688670	DQ990508	*Hamatopeduncularia cangatae*^a^ *Chauhanellus neotropicalis*
*Bagre bagre*	AY688673	DQ990523	*Hamatopeduncularia bagre*^a^
*Bagre marinus*	DQ119472	DQ990524	*Hamatopeduncularia bagre*^b^
*Genidens barbus*	FJ626167	FJ626006	*Chauhanellus* sp.^a^ *Chauhanellus boegeri*^a^
*Genidens genidens*	FJ013164	FJ013211	*Chauhanellus* sp. *Chauhanellus boegeri*
*Notarius grandicassis*	AY688671	DQ990509	*Chauhanellus neotropicalis* *Hamatopeduncularia cangatae*
*Sciades couma*	DQ990495	JN672850	*Chauhanellus boegeri* *Chauhanellus hamatopeduncularoideum* *Chauhanellus velum*
*Sciades herzbergii*	DQ990496	DQ990520	*Chauhanellus boegeri* *Chauhanellus susamlimae* *Chauhanellus velum*^a^ *Susanlimocotyle narina*^a^
*Sciades passany*	DQ990493	JN672853	*Chauhanellus neotropicalis* *Chauhanellus susamlimae*^a^ *Chauhanellus velum*
*Sciades proops*	DQ990491	DQ990518	*Chauhanellus hypenocleithrum*^a^

a Specimen subjected to molecular analysis, where sequences (partial *18S rDNA*, *ITS1*, *5.8S rDNA* and *ITS2*) were to be used for the phylogenetic and Cophylospace analysis.

b Mendoza-Franco *et al*. (2018).

Phylogenetic analyses were carried out with concatenated sequences of genes *Cytb* and *RAG2* for the hosts and *18S rDNA*, *ITS1*, *5.8S rDNA* and *ITS2* for the parasites. All sequences were aligned using MUSCLE implemented in Geneious 7.1.3 (Kearse *et al*., 2012). The evolutionary model was selected with JModelTest 2.1.1 (Darriba *et al*., 2012) based on the Akaike information criterion. Host data were treated as subpartitions of codons, and optimal evolutionary models were selected independently for each position within the codon (*Cytb* = 1st, 2nd, 3rd = GTR + G + I; *RAG2* = 1st, 2nd = GTR + I, 3rd = GTR + G). As for the parasites, the optimal evolutionary model was selected for each marker (*18S rDNA* = GTR + G; *ITS1*, *5.8S* rDNA and *ITS2* = GTR + G + I). Phylogenetic reconstruction of hosts and parasites was performed using Bayesian inference. The reconstructions followed the partitions recommended by PartitionFinder (Lanfear *et al*., 2012) implemented with MrBayes v.3.2.6 (Ronquist *et al*., 2012). Posterior probabilities were estimated from 10^6^ and 5 × 10^5^ generations for the hosts and the parasites, respectively, with 2 independent runs of 4 simultaneous Markov Chain Monte Carlo (MCMC) algorithms, sufficient to keep the average standard deviation below 0.001. Tracer v1.7 (Rambaut *et al*., 2018) was used to verify convergence and confirm the effective sample size (ESS) (i.e. ESS values > 200). The MCMC with 1000th tree was saved, diagnostic for every 1000th generation with burn-in periods set to the first 25 000 generations. Trees were visualized on Figtree 1.3.1 (Rambaut, 2008) and figures were prepared using CorelDRAW^©^ 2014.

GenBank sequences of *Ageneiosus atronasus* Eigenmann & Eigenmann, 1888 (Auchenipteridae), *Cetopsorhamdia* sp. (Heptapteridae), *Ictalurus punctatus* (Rafinesque, 1818) (Ictaluridae), *Galeichthys ater* Castelnau, 1861, *Galeichthys feliceps* Valenciennes, 1840, *Galeichthys peruvianus* Lutken, 1874 (Ariidae) (all fishes), *Gyrodactylus bueni* Bueno–Silva & Boeger, 2014 and *Gyrodactylus corydori* Bueno–Silva & Boeger, 2009 (Gyrodactylidae) (all parasites) were used as an outgroup in the reconstruction of the fish and parasite phylogenies, respectively. All sequences of the parasite species obtained for the *18S rDNA*, *ITS1*, *5.8S rDNA* and *ITS2* genes have been deposited in GenBank (Table 1).

### Morphological data from parasite and host, matrix of host–parasite associations and coevolutionary analyses

Cophylospace (Blasco-Costa *et al*., 2021) is based on the comparison of morphological and phylogenetic data from hosts and parasites to measure the strength of 3 quantitative parameters: the parasite interaction, the host interaction and the cophylogenetic signal. Parasite interaction evaluates whether phylogenetically close hosts are associated with more morphologically similar parasites than expected by chance. Conversely, host interaction evaluates whether phylogenetically close parasites are associated with more morphologically similar hosts than expected by chance, then morphological data of both hosts and parasites are required.

For parasites, we used information on the shape of dorsal and ventral anchors of the haptor. We chose these attachment structures because their morphology is likely driven by a combination of both adaptive forces and phylogenetic constraints (Mandeng *et al*., 2015; Rodríguez-González *et al*., 2016, 2017). In addition, they are not subjected to large variation due to contraction or flattening on fixation (Lim and Gibson, 2009; Rodríguez-González *et al*., 2017).

We used geometric morphometrics techniques through acquisition and landmark superimposition to characterize the shape of dorsal and ventral anchors (Klingenberg, 2010; Rodríguez-González *et al*., 2017). For acquisition of landmarks, we used the drawings of ventral and dorsal anchors of the original descriptions (Domingues and Fehlauer, 2006; Domingues *et al*., 2016; Soares *et al*., 2021). For *Chauhanellus* sp., we prepared new illustrations according to the procedures described by Domingues *et al*. (2016).

**Fig. 2.**
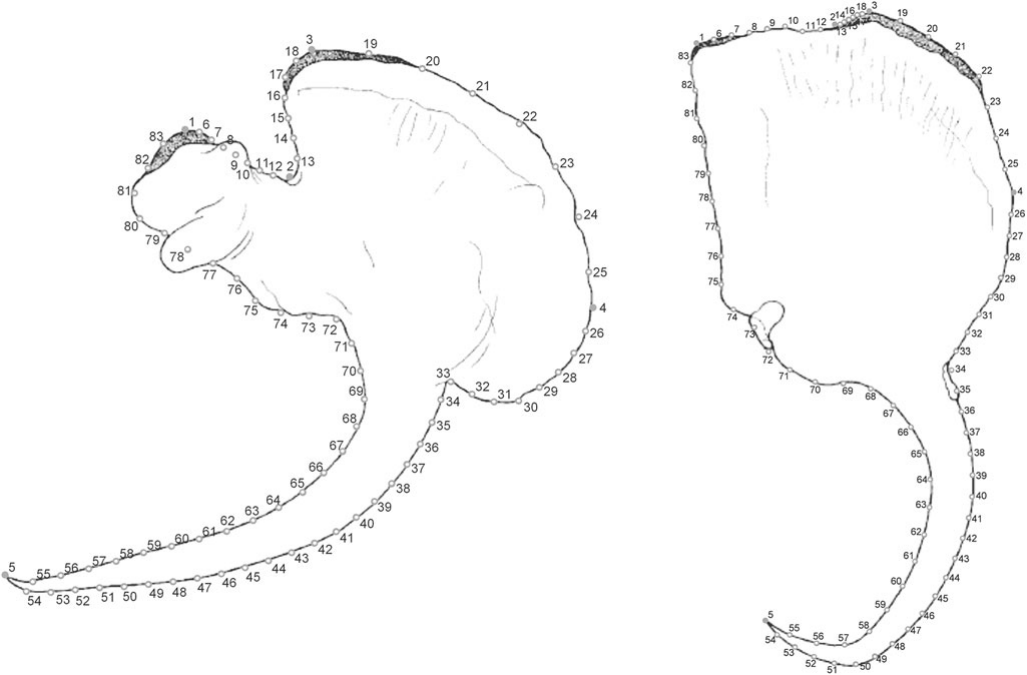
Ventral and dorsal anchors of *Chauhanellus boegeri* Domingues and Fehlauher, 2006. Distribution of landmarks (1–5, filled points) and semi-landmarks (6–83, open points) considered in the present study in ventral and dorsal anchors. Landmarks were defined as follows: 1, top of inner root; 2, inflexion between outer root and inner root; 3, top of outer root; 4, outer shaft base; 5, tip of point. Five groups of 6–29 semi-landmarks were placed equidistantly between landmark pairs as shown.

One dorsal and 1 ventral anchor of each monogenoid species were processed independently. In each anchor, 5 landmarks were placed (Llopis-Belenguer *et al*., 2015; Rodríguez-González *et al*., 2015) (Fig. 2). In addition, in order to capture anchor morphology more accurately, we employed semi-landmarks (Mitteroecker and Gunz, 2009; Llopis-Belenguer *et al*., 2015). This was appropriate in our case because of the curved inner and outer roots, and the blade and point lack of easily detectable homologous points. Five groups of 6–29 semi-landmarks were placed equidistantly between landmark pairs as shown in Fig. 2. The morphology of each anchor was defined by the Cartesian coordinates (*x*, *y*) of the 83 anatomical points (i.e. landmarks and semi-landmarks). These geometric coordinates were processed with the TPS series (Rohlf, 2022). Generalized Procrustes analysis in MorphoJ was employed to obtain matrices of shape coordinates of the dorsal and ventral anchors (datasets 1 and 2 respectively; see data availability statement below) (Klingenberg, 2011).

To test the host interaction, we produced a distance matrix based on the number of synapomorphies from a phylogenetic tree of Ariidae species built from morphological characters (e.g. mesethmoid posterior branches moderately long, delimiting between one-quarter and half of the length of the anterior cranial fontanel; lachrymal tubules differentiated from frontal bones throughout their entire extension) sensu Marceniuk *et al*. (2012*b*, p. 609) (dataset 3, Supplementary Table S1). The information on gill and nostril shape should have been preferably used as it represents the microhabitat from each parasite, but unfortunately these data are not available. Therefore, we assumed that the overall morphological similarities and differences between each host species correlate to some extent with the properties of the parasites' microhabitats.

The relationship between host and parasite phylogenies (cophylogenetic signal), and between phylogenies and morphology (parasite and host interactions) was assessed with Procrustean Approach to Cophylogeny (PACo) (Balbuena *et al*., 2013). To this end, a binary matrix describing the associations between host and parasite species [0, no association with parasite; 1, association with parasite (dataset 4)] was built based on the distribution of parasites in the hosts (Table 2) (Balbuena *et al*., 2013).

Cophylogenetic signal and interactions between phylogeny and morphology of parasites and hosts (parasite and host interaction) were measured using Procrustes *R*^2^ = 1–*m*^2^*_xy_* (Legendre and Legendre, 2012), where *m*^2^*_xy_* is the residual sum of squares produced by PACo run in symmetric mode (i.e. no *a priori* dependence of the phylogeny onto another is assumed). Both analyses were performed with package paco (Hutchinson *et al*., 2017) in R (R Core Team, 2022). The significance of the associations (at the 0.05 level) between hosts and parasites was established based on 10 000 permutations.

To evaluate the reliability of our results, we also applied Cophylospace using 1000 randomly chosen pairs of the posterior-probability trees used to build the consensus trees of parasites and hosts (excluding the burn-in set). To evaluate phylogenetic congruence between hosts and parasites, PACo was run with each of the 1000 pairs of trees, whereas for morphology–phylogeny comparisons, PACo was applied to 1000 either host or monogenoid trees and the corresponding morphology matrix. Each analysis yielded 1000 Procrustes *R*^2^, and the 95% confidence intervals (CI) were computed empirically.

To independently assess the importance of cospeciation in the association between the monogenoids and their ariid hosts, we applied Jane v4 (Conow *et al*., 2010). We used as input, host and parasite phylogenies and the matrix of host–parasite associations (dataset 5). Jane allows different costs to be set for each of the 5 coevolutionary events: cospeciation, duplication, loss, failure to diverge and duplication followed by host switching. Following Deng *et al*. (2013) and Míguez-Lozano *et al*. (2017), 20 event cost schemes were applied (Table 3), under 1000 generations and a population size of 200 as parameters of Jane's genetic algorithm. Since our intention was to establish whether cospeciation events are important in the system studied, the cost of cospeciation was not to be larger than that of duplication, host-switch or loss (Charleston and Libeskind-Hadas, 2014), and thus it was set to 0 in all analyses. The statistical significance (at the 0.05 level) of global cost tests was assessed using 1000 randomizations under Random Tip Mapping.

**Table 3.** Results of cophylogenetic analyses with Jane for monogenoids and their ariid hosts

Model	Event costs	Total cost	Cospeciation	Duplication	Duplication and host switch	Loss	Failure to diverge
Jane default	01211	45	2	5	2	22	14
TreeMap default	01111	43	2	5	2	22	14
TreeFitter default	00211	39	2	6	1	23	14
Host switch-adjusted TreeFitter	00111	38	1–2	5–6	2	22	14
DHS > or = D^a^	00010	22	1	5–6	2–3	22	14
DHS > or = D	00011	36	1	5–6	2–3	22	14
DHS > or = D	00012	50	1	5–6	2–3	22	14
DHS > or = D	00020	44	1	5–6	2–3	22	14
DHS > or = D	00021	58	1	5–6	2–3	22	14
DHS > or = D	00022	72	1	5–6	2–3	22	14
DHS > or = D	00110	24	1–2	5–6	2	22	14
DHS > or = D	00112	52	1–2	5–6	2	22	14
DHS > or = D	00120	46	1–2	5–6	2	22	14
DHS > or = D	00121	60	1–2	5–6	2	22	14
DHS > or = D	00122	74	1–2	5–6	2	22	14
DHS > or = D	01110	29	2	5	2	22	14
DHS > or = D	01112	57	2	5	2	22	14
DHS > or = D	01120	51	2	5	2	22	14
DHS > or = D	01121	79	2	5	2	22	14
DHS > or = D	01122	65	2	5	2	22	14

Twenty evolutionary models with different cost schemes for each evolutionary event were tested. The total costs and frequencies of individual evolutionary events are shown for each model. The statistical significance of global cost tests (*P* < 0.05) was established based on 1000 random reconstructions. All models were significant.

a Míguez-Lozano *et al*. (2017), DHS > or = D = cost of duplication followed by host-switch to be higher, or equal to cost of duplication.

## Results

The host–parasite system examined is composed of 10 monogenoid species on 12 South American species of marine catfish (Ariidae) (Table 2, Fig. 1). The aligned host sequence lengths were 720 pb for partial *Cytb* and 1095 pb for partial *RAG2* and those of the parasites were 1547 pb for partial *18S rDNA*, *ITS1*, *5.8S rDNA* and *ITS2*.

**Fig. 3.**
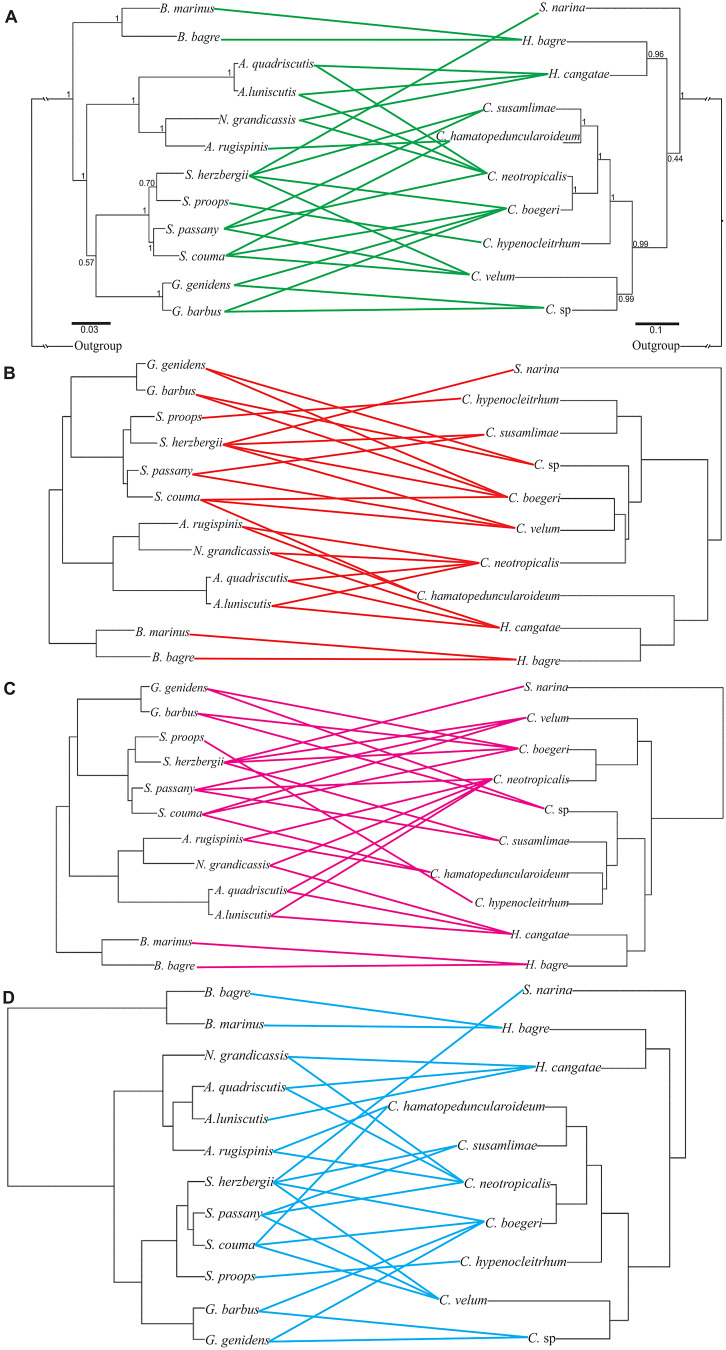
Tanglegram reflecting the application of the Cophylospace Framework. (A) Associations between 12 species of ariids from Atlantic coast of South America (left) and 10 species of monogenoids (right) – support values of posterior probabilities are given above the branches. (B) Interaction of host phylogeny with monogenoid shape (1) – association between the phylogeny of 12 ariid species and the shape of the ventral anchors of 10 species of monogenoids. (C) Interaction of host phylogeny with monogenoid shape (2) – association between the phylogeny of 12 ariid species and the shape of the dorsal anchors of 10 species of monogenoids. (D) Interaction of monogenoids phylogeny with ariid shape – association between morphological characters of 12 ariid species and the phylogeny of 10 species of monogenoids. Species names are the same as in Table 2.

In general, posterior probabilities indicated strong nodal support in both the host and parasite phylogenies (Fig. 3A). Exceptions in the fish phylogeny were nodes between *Genidens* and *Sciades* (0.57) and between *S. herzbergii* and *S. proops* (0.70). For the parasites, only the node between the clade of *Hamatopeduncularia* spp. [*H. bagre* from *B. marinus* (Mitchill, 1815) and *B. bagre*, *H. cangatae* from *A. quadriscutis*, *A. luniscutis* and *N. grandicassis*] and *Chauhanellus* spp. parasitizing all other ariids showed relatively low support (0.44) (Fig. 3A).

**Fig. 4.**
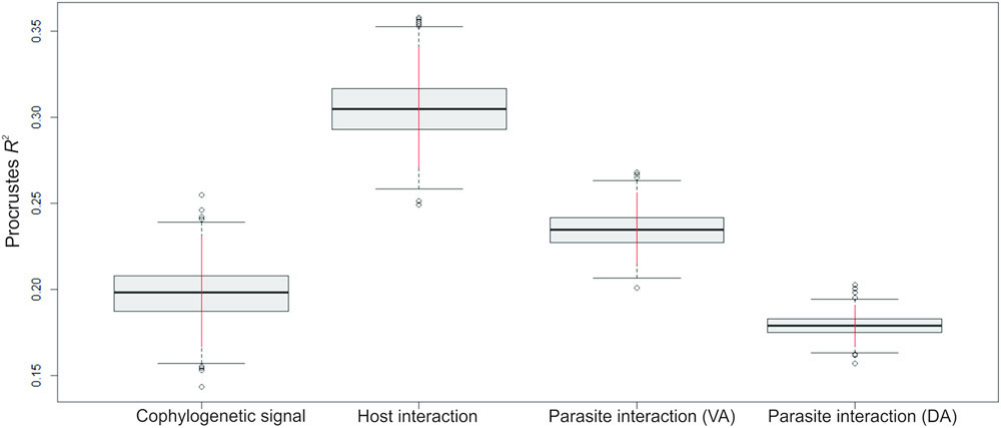
Boxplots and 95% confidence intervals (red bar) of the Procrustes *R*^2^ estimated with the sets of posterior probability trees of interaction. Cophylogenetic signal = strength of the relationship between phylogenies of the ariids and monogenoids; host interaction = strength of the relationship between morphology of ariid species and the phylogeny of monogenoids; parasite interaction = strength of the relationship between the ariid phylogeny with differences in the shape of monogenoids' anchors (VA, ventral anchor; DA, dorsal anchor).

The analysis revealed a low, but statistically significant, cophylogenetic signal (*R*^2^ = 0.20, *P* = 0.018) (Fig. 3A). The tests examining the interaction of ariids phylogeny with the shape of ventral and dorsal anchors of monogenoids (parasite interaction) yielded Procrustes *R*^2^ = 0.24 and *R*^2^ = 0.18, respectively (Fig. 3B and C, respectively). This host phylogeny–parasite morphology interaction was significant for the ventral anchors (*P* = 0.004), but not for the dorsal ones (*P* = 0.055). The concordance between monogenoid phylogeny and ariid shape (host interaction) was higher (*R*^2^ = 0.30) and highly significant (*P* = 0.001) (Fig. 3D). Figure 4 shows the boxplots and 95% CI of the *R*^2^ estimated with the sets of post-probability trees, suggesting that the pattern of highest *R*^2^ associated with the host morphology–parasite phylogeny interaction is not critically affected by phylogenetic uncertainty.

With all 20-cost schemes, every scenario led to a significant global cost (*P* < 0.05). Table 3 displays the number of coevolutionary events under the different cost models. Loss and failure to diverge were the most common coevolutionary events in the ariid–monogenoid system studied, whereas cospeciation was the least recurrent event (1–2) under all cost schemes.

## Discussion

The parasite phylogeny obtained in this study was constructed based on unpublished molecular data (partial *18S rDNA*, *ITS1*, *5.8S rDNA* and *ITS2*) and represents the first complete phylogenetic hypothesis of monogenoids parasitizing ariids from South America. Our analyses showed that *S. narina*, *Hamatopeduncularia* spp. and *Chauhanellus* spp. form a monophyletic group. This suggests that these parasites colonized the hosts only once in their evolutionary history, followed by diversification (Fig. 3A) (Soares *et al*., 2021). However, despite the clades formed by *Hamadopeduncularia* spp. and *Chauhanellus* spp. being well supported, the relationships between the 2 genera had low support (Fig. 3A).

As for the phylogenetic reconstruction of the hosts, the overall relationships at genus and species level agree with Marceniuk *et al*. (2012*b*) (morphological data) and Marceniuk *et al*. (2019*b*) (morphological and molecular data). The low support of the node separating *Genidens* and *Sciades* also conforms with Marceniuk *et al*. (2012*b*, 2019*b*). Therefore, despite the low support of some of the clades, the phylogenies obtained were considered reliable enough for Cophylospace assessment (Blasco-Costa *et al*., 2021).

The Cophylospace analyses showed that cophylogenetic signal was statistically significant in the ariid‒monogenoid system. A significant cophylogenetic signal has been often interpreted as offering support for cospeciation (Desdevises *et al*., 2002; Šimková *et al*., 2004, 2006, 2013; Huyse and Volckaert, 2005; Mendlová *et al*., 2012; Hahn *et al*., 2015; Vanhove *et al*., 2015; Míguez-Lozano *et al*., 2017; Graça *et al*., 2018). However, previous studies suggest that cospeciation is not an important driver in the evolution of monogenoids (Desdevises *et al*., 2002; Šimková *et al*., 2004, 2013; Blasco-Costa *et al*., 2012; Míguez-Lozano *et al*., 2017). This seems to apply to our system. In all scenarios suggested by Jane, our analyses identified only 1–2 cases of cospeciation out of 22–75 evolutionary events indicated by different cost schemes (Table 3, Supplementary Fig. S1), which is in line with the results of the Cophylospace analysis. Whereas phylogenetic and morphological distance of monogenoids contributed similarly to explain the pattern of host–parasite associations, parasite phylogeny was more strongly associated with the morphological traits of the hosts than with the host phylogeny, as the respective CIs do not overlap (Fig. 4).

In fact, the position of ariid‒monogenoid systems in the Cophylospace (Supplementary Fig. S2) suggests some degree of asymmetry in which host morphological traits may have influenced diversification of their monogenoids, supporting that the speciation in our system is driven to a larger extent by phylogenetic tracking of host resources rather than by cospeciation. Thus, host morphological traits driven by adaptive processes linked to their occupation of different environments in South America in parallel seem to be the main force that has driven the speciation in these parasites. This agrees with a coadaptive codiversification scenario sensu Clayton *et al*. (2015). Our results also conform with those reported by Blasco–costa *et al*. (2021) for monogenoids (*Ligophorus* spp.) on grey mullets (Mugilidae) in the Mediterranean and the Black Sea.

It is known that the host can drive genetic and morphological differentiation of monogenoids (Desdevises *et al*., 2002) and it has been often hypothesized that haptor morphology reflects adaptations for attachment to the host (Šimková *et al*., 2002; Mandeng *et al*., 2015; Rodríguez-González *et al*., 2016, 2017). Indeed, the second largest characteristic interaction force in our system is linked to the shape of the ventral anchors (Fig. 4), followed by the cophylogenetic signal, reinforcing our hypothesis of speciation by adaptation.

Monogenoids are known to be highly specific to their hosts (Poulin, 1992; Braga *et al*., 2014). However, this specificity does not necessarily have to result from cospeciation of host and parasite lineages. Other processes, such as duplication and host-switching (Boeger and Kritsky, 1997; Braga *et al*., 2014) or failure to diverge and losses, can result in host specificity (Vanhove *et al*., 2015; Míguez-Lozano *et al*., 2017). More generally, it has been proposed that parasites do not specialize in particular host species, but in resources that can or cannot be, on an evolutionary scale, shared among a range of host species (Nyman, 2010). It has also been suggested that parasites, in addition to specialists and generalists, can be false specialists or false generalists (Brooks and McLennan, 2002). False specialists are generalists restricted to a single or few resources due to ecological or temporary factors (opportunity), whereas false generalists are specialized in a resource that is phylogenetically widespread (phylogenetically closely host species with similar ecological requirements) (Brooks and McLennan, 2002). The latter seems to be the case in the present study and in other host‒monogenoid systems (Desdevises *et al*., 2002; Mendlová *et al*., 2012; Braga *et al*., 2014; Wendt *et al*., 2022). Further studies should examine to which extent the false generalist paradigm applies to monogenoids and other ectoparasites.

Although we acknowledge that information on the shape of the gills and nostrils of each host is needed to better assess the relationships between the occupation of each microhabitat by each monogenoid species, the Cophylospace Framework supplies a quantitative tool that not only supports that cospeciation is not a major driver of coevolutionary relations between monogenoids and ariids, but also allows quantifying the strength of different drivers of host–parasite coevolution (cophylogenetic signal, host interaction and parasite interaction). However, we suggest that future research based on the Cophylospace Framework would greatly benefit from more detailed morphological studies of the hosts.

## Data Availability

Datasets 1–5 required to perform all the analyses are deposited on Zenodo (https://doi.org/10.5281/zenodo.7074210).

## Supplementary material

For supplementary material accompanying this paper visit https://doi.org/10.1017/S0031182022001615. click here to view supplementary material
