# Supplementary material for: Diversification processes between monogenoids (Dactylogyridae) and their marine catfish (Siluriformes: Ariidae) from the Atlantic coast of South America
Source: Parasitology. 2022 Nov 29;150(2):184–94. doi: 10.1017/S0031182022001615 (PMC10106279; doi:10.1017/S0031182022001615)
Supplement: Supplementary file 1 [file S0031182022001615sup001.zip › S0031182022001615sup001.docx]

Diversification process between monogenoids (Dactylogyridae) and their marine catfish (Siluriformes: Ariidae) from the Atlantic coast of South America

G.B. Soares, E.A. Adriano, M.V. Domingues, J.A. Balbuena

Address correspondence to [j.a.balbuena@uv.es](mailto:j.a.balbuena@uv.es)


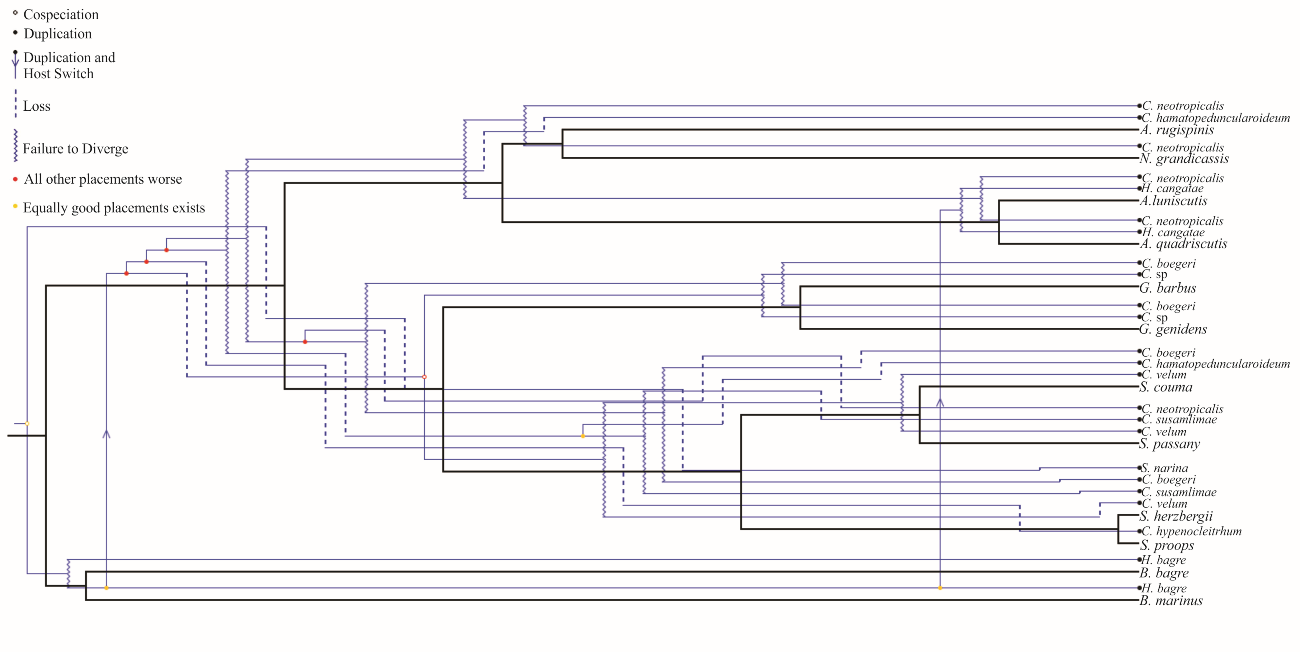


Figure S1. One of the possible cophylogenetic scenarios of monogenoids and their ariid hosts returned by Jane (2 cospeciation events, 5 duplications, 2 duplications followed by host switch, 14 failures to diverge and 22 losses) produced with the default cost values of Jane. Black branches (independent) represent the host phylogeny. Blue branches (dependent) correspond to the parasite phylogeny. Species abbreviations are the same as in Table 2.


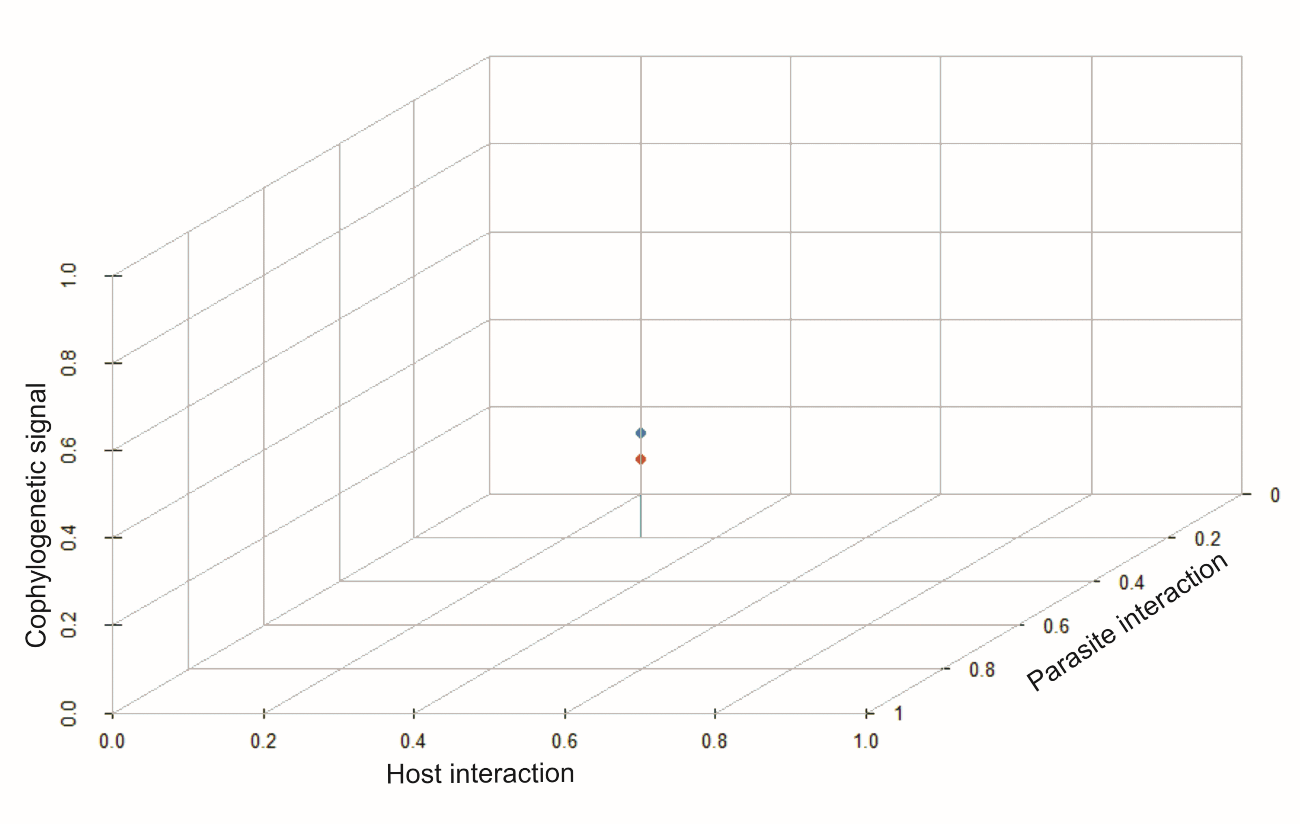


Figure S2. Position in Cophylospace of the host–parasite system studied (twelve ariid species associated with 10 species of monogenoids). The axes represent Procrustes *R^2^* = 1 – *m*^2^. Blue point = Interaction based with shape of the ventral anchors; red point = Interaction based with shape of the dorsal anchors. Cophylogenetic signal, host interaction and parasite interaction are the same as in Figure 4.
