# Supplementary material for: Diversification processes between monogenoids (Dactylogyridae) and their marine catfish (Siluriformes: Ariidae) from the Atlantic coast of South America
Source: Parasitology. 2022 Nov 29;150(2):184–94. doi: 10.1017/S0031182022001615 (PMC10106279; doi:10.1017/S0031182022001615)
Supplement: Supplementary file 1 [file S0031182022001615sup001.zip › S0031182022001615sup002.docx]

# Suplementary S1

# Diversification process between monogenoids (Dactylogyridae) and their marine

# catfish (Siluriformes: Ariidae) from the Atlantic coast of South America

# G.B. Soares, E.A. Adriano, M.V. Domingues, J.A. Balbuena

# Address correspondence to j.a.balbuena@uv.es

# R script to Compute Cophylospace R2 (Blasco-Costa et al. 2021)

# Input files available on Zenodo https://doi.org/10.5281/zenodo.7074210

#

##### Script starts here #######################################################

# Load libraries required

library**(**paco**)**

library**(**phytools**)**

# 1. COMPUTE PROCRUSTES R2 IN EACH COPHYLOSPACE DIMENSION

# 1.1. COMPARISON PHYLOGENY H - PHYLOGENY P

# Read trees

Htree **<-** read.tree**(**"host_tree.txt"**)**

Ptree **<-** read.tree**(**"parasite_tree.txt"**)**

# Read HP assoc matrix

HP **<-** read.table**(**"Dataset_4_matrix_Parasite_Host_association.txt",

header **=** **TRUE**, row.names **=** 1**)**

HP **<-** as.matrix**(**HP**)**

# Compute patristic distances

DH **<-** cophenetic**(**Htree**)**

DP **<-** cophenetic**(**Ptree**)**

D **<-** prepare_paco_data**(**DH, DP, HP**)**

D **<-** add_pcoord**(**D**)**

D **<-** PACo**(**D, nperm **=** 10000, method**=**"r0", symmetric **=** **TRUE**,

proc.warnings **=** **TRUE**, shuffled **=** **TRUE)**

print**(**D**$**gof**)**

R2a **<-** 1 **-** D**$**gof**$**ss

print**(**R2a**)**

# 1.2. COMPARISON PHYLOGENY P - MORPHOLOGY H

HMtree **<-** read.tree**(**"Dataset_3_matrixdistance_of_host_morfo_Marceniuk2012.txt"**)**

Ptree **<-** read.tree**(**"parasite_tree.txt"**)**

DH **<-** cophenetic**(**HMtree**)**

DP **<-** cophenetic**(**Ptree**)**

D **<-** prepare_paco_data**(**DH, DP, HP**)**

D **<-** add_pcoord**(**D**)**

D **<-** PACo**(**D, nperm **=** 10000, method**=** "r0", symmetric **=** **TRUE**,

proc.warnings **=** **TRUE**, shuffled **=** **TRUE)**

print**(**D**$**gof**)**

R2HM **<-** 1 **-** D**$**gof**$**ss

print**(**R2HM**)**

# 1.3. COMPARISON PHYLOGENY H - MORPHOLOGY P

# 1.3.1 VENTRAL ANCHORS

Htree **<-** read.tree**(**"host_tree.txt"**)**

ProAVtree **<-** read.tree**(**"Dataset_1_matrix_shape_coordinates_Anchor_Ventral.txt"**)**

DH **<-** cophenetic**(**Htree**)**

DP **<-** cophenetic**(**ProAVtree**)**

D **<-** prepare_paco_data**(**DH, DP, HP**)**

D **<-** add_pcoord**(**D**)**

D **<-** PACo**(**D, nperm **=** 10000, method **=** "r0", symmetric **=** **TRUE**,

proc.warnings **=** **TRUE**, shuffled **=** **TRUE)**

print**(**D**$**gof**)**

R2ProAV **<-** 1 **-** D**$**gof**$**ss

print**(**R2ProAV**)**

# 1.3.2 DORSAL ANCHORS

Htree **<-** read.tree**(**"host_tree.txt"**)**

ProADtree **<-** read.tree**(**"Dataset_2_matrix_shape_coordinates_Anchor_Dorsal.txt"**)**

DH **<-** cophenetic**(**Htree**)**

DP **<-** cophenetic**(**ProADtree**)**

D **<-** prepare_paco_data**(**DH, DP, HP**)**

D **<-** add_pcoord**(**D**)**

D **<-** add_pcoord**(**D, correction **=** **)**

D **<-** PACo**(**D, nperm **=** 10000, method **=** "r0", symmetric **=** **TRUE**,

proc.warnings **=** **TRUE**, shuffled **=** **TRUE)**

print**(**D**$**gof**)**

R2ProAD **<-** 1 **-** D**$**gof**$**ss

print**(**R2ProAD**)**

# 2. COMPUTE PROCRUSTES R2 AND CONFIDENCE INTERVALS BASED ON

# POST-PROBABILITY TREES

## Read post-probability trees; parasites and hosts

Ptrees **<-** read.nexus**(**"parasite_Tree_run1000tree.t"**)**

Ptrees **<-** Ptrees**[-**c**(**1,502**)]** # remove burn in set

# Trees need to be rooted correctly

Ptrees **<-** lapply**(**Ptrees, **function(**x**)** ape**::**root**(**x, outgroup **=** c**(**"G_bu", "G_co"**)))**

class**(**Ptrees**)** **<-** "multiPhylo"

# Root taxa dropped

Ptrees **<-** lapply**(**Ptrees, **function(**x**)** ape**::**drop.tip**(**x, tip **=** c**(**"G_bu", "G_co"**)))**

class**(**Ptrees**)** **<-** "multiPhylo"

Htrees **<-** read.nexus**(**"host_Tree_run2000tree.t"**)**

Htrees **<-** Htrees**[-**c**(**1,502**)]** # remove burn in set

# Trees need to be rooted correctly

# We tried to root trees at c("G_at", "G_fe", "G_pe", "I_pu", "C_sp", "A_at")

# but some taxa were not always at the root

# Since the consensus tree shows "A_at" at root, trees were rooted as follows

Htrees **<-** lapply**(**Htrees, **function** **(**x**)** ape**::**root**(**x, outgroup **=**"A_at"**))**

class**(**Htrees**)** **<-** "multiPhylo"

# Root taxa dropped

Htrees **<-** lapply**(**Htrees, **function** **(**x**)** ape**::**drop.tip**(**x,

tip **=** c**(**"G_at", "G_fe", "G_pe", "I_pu", "C_sp", "A_at"**)))**

class**(**Ptrees**)** **<-** "multiPhylo"

# There are 2000 host trees. Select 1000 randomly

selectT **<-** sample**(**1**:**2000, 1000**)**

Htrees **<-** Htrees**[**selectT**]**

# Compute patristic distances post-probability trees

DisH **<-** lapply**(**Htrees, cophenetic**)**

DisP **<-** lapply**(**Ptrees, cophenetic**)**

# 4.1 PHYLOGENY H - PHYLOGENY P

# Compute R2 post-probability trees

R2 **<-** rep**(NA**, length**(**DisH**))**

**for(**i **in** 1**:**length**(**DisH**))** **{**

D **<-** prepare_paco_data**(**DisH**[[**i**]]**, DisP**[[**i**]]**, HP**)**

D **<-** add_pcoord**(**D**)**

D **<-** PACo**(**D, nperm **=** 1, method **=** "r0", symmetric **=** **TRUE)**

R2**[**i**]** **<-** 1 **-** D**$**gof**$**ss

**}**

# 4.2 PHYLOGENY P - MORPHOLOGY H

# Read morphology data

MH **<-** read.tree**(**"Dataset_3_matrixdistance_of_host_morfo_Marceniuk2012.txt"**)**

DMH **<-** cophenetic**(**MH**)**

# Compute R2s

R2MH **<-** rep**(NA**, length**(**DisH**))**

**for(**i **in** 1**:**length**(**DisH**))** **{**

D **<-** prepare_paco_data**(**DMH, DisP**[[**i**]]**, HP**)**

D **<-** add_pcoord**(**D**)**

D **<-** PACo**(**D, nperm**=**1, method**=**"r0", symmetric **=** **TRUE)**

R2MH**[**i**]** **<-** 1 **-** D**$**gof**$**ss

**}**

D **<-** prepare_paco_data**(**DMH, DP, HP**)**

D **<-** add_pcoord**(**D**)**

D **<-** PACo**(**D, nperm**=**1, method**=**"r0", symmetric **=** **TRUE)**

R2CMH **<-** 1 **-** D**$**gof**$**ss

# 4.3 PHYLOGENY H - MORPHOLOGY P

# 4.3.1 VENTRAL ANCHORS

# Read P morphological data

MPv **<-** read.tree**(**"Dataset_1_matrix_shape_coordinates_Anchor_Ventral.txt"**)**

DMPv **<-** cophenetic**(**MPv**)**

#Compute R2s

R2MPv **<-** rep**(NA**, length**(**DisH**))**

**for(**i **in** 1**:**length**(**DisH**))** **{**

D **<-** prepare_paco_data**(**DisH**[[**i**]]**, DMPv, HP**)**

D **<-** add_pcoord**(**D**)**

D **<-** PACo**(**D, nperm**=**1, method**=**"r0", symmetric **=** **TRUE)**

R2MPv**[**i**]** **<-** 1 **-** D**$**gof**$**ss

**}**

D **<-** prepare_paco_data**(**DH, DMPv, HP**)**

D **<-** add_pcoord**(**D**)**

D **<-** PACo**(**D, nperm**=**1, method**=**"r0", symmetric **=** **TRUE)**

R2CMPv **<-** 1 **-** D**$**gof**$**ss

# 4.3.2 DORSAL ANCHORS

# Read P morphological data

MPd **<-** read.tree**(**"Dataset_2_matrix_shape_coordinates_Anchor_Dorsal.txt"**)**

DMPd **<-** cophenetic**(**MPd**)**

# Compute R2s

R2MPd **<-** rep**(NA**, length**(**DisH**))**

**for(**i **in** 1**:**length**(**DisH**))** **{**

D **<-** prepare_paco_data**(**DisH**[[**i**]]**, DMPd, HP**)**

D **<-** add_pcoord**(**D**)**

D **<-** PACo**(**D, nperm**=**1, method**=**"r0", symmetric **=** **TRUE)**

R2MPd**[**i**]** **<-** 1 **-** D**$**gof**$**ss

**}**

D **<-** prepare_paco_data**(**DH, DMPd, HP**)**

D **<-** add_pcoord**(**D**)**

D **<-** PACo**(**D, nperm**=**1, method**=**"r0", symmetric **=** **TRUE)**

R2CMPd **<-** 1 **-** D**$**gof**$**ss

# Plot results

boxplot**(**R2, R2MH, R2MPv, R2MPd, names **=** c**(**"Cophylogenetic signal",

"Host interaction",

"Parasite interaction (VA)",

"Parasite interaction (DA)"**)**,

ylab **=** "Procrustes R2"**)**

lines**(**c**(**1, 1**)**, quantile**(**R2, c**(**0.025, 0.975**))**, col **=**"red"**)**

lines**(**c**(**2, 2**)**, quantile**(**R2MH, c**(**0.025, 0.975**))**, col **=**"red"**)**

lines**(**c**(**3, 3**)**, quantile**(**R2MPv, c**(**0.025, 0.975**))**, col **=**"red"**)**

lines**(**c**(**4, 4**)**, quantile**(**R2MPd, c**(**0.025, 0.975**))**, col **=**"red"**)**

#Script ends here #############################################################

# REFERENCE

# Blasco-Costa et al. 2021. Trends in Ecology & Evolution 36: 907-918

# https://doi.org/10.1016/j.tree.2021.06.006
