# Supplementary material for: Diversification processes between monogenoids (Dactylogyridae) and their marine catfish (Siluriformes: Ariidae) from the Atlantic coast of South America
Source: Parasitology. 2022 Nov 29;150(2):184–94. doi: 10.1017/S0031182022001615 (PMC10106279; doi:10.1017/S0031182022001615)
Supplement: Supplementary file 1 [file S0031182022001615sup001.zip › S0031182022001615sup003.docx]

Diversification process between monogenoids (Dactylogyridae) and their marine catfish (Siluriformes: Ariidae) from the Atlantic coast of South America

G.B. Soares, E.A. Adriano, M.V. Domingues, J.A. Balbuena

Address correspondence to [j.a.balbuena@uv.es](mailto:j.a.balbuena@uv.es)

Table S1. Additional details on the construction of the distance matrix of host species based on morphological characters sensu Marceniuk et al. (2012b).

|  | **B_ba** | **B_ma** | **S_co** | **S_pr** | **S_he** | **S_pa** | **G_ge** | **G_ba** | **A_ru** | **N_gr** | **A_lu** | **A_qu** |
| --- | --- | --- | --- | --- | --- | --- | --- | --- | --- | --- | --- | --- |
| **B_ba** | 0 | 46 | 137 | 146 | 137 | 139 | 122 | 122 | 155 | 139 | 157 | 157 |
| **B_ma** | 46 | 0 | 137 | 146 | 137 | 139 | 122 | 122 | 155 | 139 | 157 | 157 |
| **S_co** | 137 | 137 | 0 | 27 | 16 | 11 | 51 | 51 | 76 | 60 | 78 | 78 |
| **S_pr** | 146 | 146 | 27 | 0 | 27 | 25 | 60 | 60 | 85 | 69 | 87 | 87 |
| **S_he** | 137 | 137 | 16 | 27 | 0 | 21 | 51 | 51 | 76 | 60 | 78 | 78 |
| **S_pa** | 139 | 139 | 11 | 25 | 21 | 0 | 54 | 54 | 79 | 63 | 81 | 81 |
| **G_ge** | 122 | 122 | 51 | 60 | 51 | 54 | 0 | 12 | 69 | 53 | 71 | 71 |
| **G_ba** | 122 | 122 | 51 | 60 | 51 | 54 | 12 | 0 | 69 | 53 | 71 | 71 |
| **A_ru** | 155 | 155 | 76 | 85 | 76 | 79 | 69 | 69 | 0 | 34 | 35 | 35 |
| **N_gr** | 139 | 139 | 60 | 69 | 60 | 63 | 53 | 53 | 34 | 0 | 36 | 36 |
| **A_lu** | 157 | 157 | 78 | 87 | 78 | 81 | 71 | 71 | 35 | 36 | 0 | 14 |
| **A_qu** | 157 | 157 | 78 | 87 | 78 | 81 | 71 | 71 | 35 | 36 | 14 | 0 |
| Labels: B_ba = *Bagre bagre*; B_ma = *Bagre marinus*; S_co = *Sciades couma*; S_pr = *Sciades proops*; S_he = *Sciades herbergii*; S_pa = *Sciades passany*; G_ge = *Genidens genidens*; G_ba = *Genidens barbus*; A_ru = *Amphiarius rugispinis*; N_gr = *Notarius grandicassis*; A_lu = *Aspistor luniscutis*; A_qu = *Aspistor quadriscutis.* | | | | | | | | | | | | |

The matrix was constructed as follows:

*Bagre bagre* and *B. marinus* are sister groups (see Marceniuk et al. [2012 b], Clade 6, figure 29, p. 607). These species share characteristics with Clades 4 and 5. The patristic distance between *Bagre bagre* and *B. marinus* are the sum of these characteristics. Clade 4 has 39 synapormorphies, Clade 5, 4 and Clade 6, 3 (39 + 4 + 3 = 46). For the list of morphological characters see Marceniuk *et al.* (2012 b, pp. 606-635).
